# Assessment and management of heart failure in patients with chronic kidney disease

**DOI:** 10.1007/s10741-023-10346-x

**Published:** 2023-09-20

**Authors:** Andrea Igoren Guaricci, Francesca Sturdà, Roberto Russo, Paolo Basile, Andrea Baggiano, Saima Mushtaq, Laura Fusini, Fabio Fazzari, Fulvio Bertandino, Francesco Monitillo, Maria Cristina Carella, Marco Simonini, Gianluca Pontone, Marco Matteo Ciccone, Giuseppe Grandaliano, Giuseppe Vezzoli, Francesco Pesce

**Affiliations:** 1 University Cardiologic Unit, Interdisciplinary Department of Medicine, Polyclinic University Hospital, University of Bari Aldo Moro, Piazza Giulio Cesare 11, 70121 Bari, Italy; 2 Department of Precision and Regenerative Medicine and Ionian Area, University of Bari Aldo Moro, 70124 Bari, Italy; 3 Department of Perioperative Cardiology and Cardiovascular Imaging, Centro Cardiologico Monzino, IRCCS, 20138 Milan, Italy; 4 Nephrology and Dialysis Unit, IRCCS San Raffaele Scientific Institute, 20132 Milan, Italy; 5 Department of Medical and Surgical Sciences, Fondazione Policlinico Universitario A. Gemelli IRCCS, Rome, Italy; 6 Department of Nephrology and Dialysis, Vita Salute San Raffaele University, 20132 Milan, Italy

**Keywords:** Heart failure, Chronic kidney disease, Biomarkers, Cardio-renal syndrome, Guidelines-directed medical therapy

## Abstract

Heart failure (HF) and chronic kidney disease (CKD) are two pathological conditions with a high prevalence in the general population. When they coexist in the same patient, a strict interplay between them is observed, such that patients affected require a clinical multidisciplinary and personalized management. The diagnosis of HF and CKD relies on signs and symptoms of the patient but several additional tools, such as blood-based biomarkers and imaging techniques, are needed to clarify and discriminate the main characteristics of these diseases. Improved survival due to new recommended drugs in HF has increasingly challenged physicians to manage patients with multiple diseases, especially in case of CKD. However, the safe administration of these drugs in patients with HF and CKD is often challenging. Knowing up to which values ​​of creatinine or renal clearance each drug can be administered is fundamental. With this review we sought to give an insight on this sizable and complex topic, in order to get clearer ideas and a more precise reference about the diagnostic assessment and therapeutic management of HF and CKD.

## Introduction

The management of heart failure (HF) has greatly improved during the recent years, but the prognosis remains poor [1]. Moreover, population aging and an increased survival after myocardial infarction are two factors that have changed patients’ profiles [2]. HF is often a concurrent condition in elderly patients with many prognosis-relevant comorbidities, such as diabetes mellitus, lung diseases, and vascular diseases. Among these, one of the most frequent is chronic kidney disease (CKD). Furthermore, HF and CKD share the same risk factors such as atherosclerosis, hypertension, diabetes mellitus, obesity, tobacco use, dyslipidemia, negatively affecting the function of both organs. They also have in common the same pathophysiological bidirectional pathways, such as endothelial dysfunction, sympathetic neurohormonal activation, inflammation, and oxidative stress. These processes converge and promote, over time, the dysfunction of both organs [3]. Therefore, the diagnostic and therapeutic management of this subset of patients are particularly challenging, requiring a multidisciplinary and personalized approach to minimize disease progression and maximize efficacy and safety of the therapeutic options.

## Epidemiology

Different meta-analyses of randomized controlled studies have shown that 49% of patients with HF suffered from CKD, with eGFR values below 60 mL/min/1.73 m^2^ [4]. In the Atherosclerosis Risk In Communities (ARIC) study, the incidence of HF was threefold higher in people with an eGFR < 60 mL/min/1.73 m^2^ compared to those with a preserved kidney function [5]. The Acute Decompensated Heart Failure National Registry (ADHERE) points out that approximately 30% of patients admitted to hospital for acute decompensated HF are affected by acute or chronic kidney disease [6, 7]. The prevalence of CKD was higher in acute HF patients (53%) compared with chronic HF patients (42%). Kidney impairment is a predictor of a poor prognosis in patients with HF and it has a strong association with worse outcomes [8]. In fact, CKD is related to a higher mortality risk in patient with HF [hazard ratio (HR) 2.34–95% confidence interval (CI) 2.20–2.50] [9].

## The cardio-renal syndrome

The cardio-renal syndrome (CRS) represents a disorder of the heart and kidney characterized by the onset of acute or chronic dysfunction in one organ which promote the acute or chronic dysfunction of the other [10].

Several mechanisms are involved in the development of CRS, such as hemodynamic alterations, neurohormonal dysregulation, inflammatory activation, fibrosis, endothelial dysfunction, atherosclerosis, and, above all, arterial stiffness, a pathological alteration of the vascular wall. All the aforementioned conditions trigger a vicious circle with mutual damage leading to the progression of cardiac and kidney disease [11].

Löfman et al. in 2017 showed that in HF with a preserved ejection fraction (HFpEF), CKD was more common and with a better prognosis than HF with a reduced ejection fraction (HFrEF) and HF with a mildly reduced ejection fraction (HFmrEF) [12]. In HFpEF co-morbidity, such as CKD, play a pivotal role, inducing an inflammatory state with microvascular dysfunction potentially leading to both cardiac and renal fibrosis. It has been proposed that HFpEF patients are more susceptible to low blood pressure, being more preload dependent and having more autonomic dysfunction with ventricular and arterial stiffness [12]. The renal hypoperfusion in HF induces the activation of the renin-angiotensin-aldosterone system (RAAS) together with sympathetic nervous system, leading to renal and systemic vasoconstriction, which in turn promotes the secretion of vasopressin. Accordingly, the fluid overload leads to renal interstitial hypertension with necrosis of tubular epithelium, tubular hypertrophy, fibrosis, and permanent tubular injury, increasing central venous pressure and venous return of right ventricle, oxidative stress, the production of proinflammatory cytokines, which exacerbate renal and cardiac remodeling through profibrotic mechanisms, finally causing a worsening of cardiac and kidney functions [12]. This situation leads to the increase of peripheral systemic resistance, arterial stiffness, filling pressures, and the thickening of left ventricle myocardial walls.

## Biomarkers

The diagnosis of HF and CKD relies on signs and symptoms, together with cardiac and renal anomalies either structural or functional. Blood biomarkers add further information on the cardiac and kidney damage, given their diagnostic, prognostic, and predictive role. As regard the cardiac evaluation, plasma concentrations of natriuretic peptides such as B type natriuretic peptide (BNP) or N-terminal pro-B-type natriuretic peptide (NT-pro-BNP) are recommended as initial diagnostic test in patients with clinical suspicion of HF to rule out the diagnosis [13, 14]. Natriuretic peptides are mainly synthesized and secreted by myocytes of the left ventricle as a response to the stretch of myocardial walls caused by pressure overload or volume expansion. NT-pro-BNP has a limited diagnostic utility due to high between-person variation related to the influence of several pathophysiological factors on its clearance (hydration status, residual renal function, dialysis regimens), which may hamper the identification of a diagnostic cut-off value in end-stage kidney disease on dialysis [15]. The within-person variability of NT-pro-BNP values is less pronounced, suggesting that a relative-change strategy may improve NT-proBNP diagnostic utility in this population. However, serial NT-proBNP concentrations need to double or halve to confidently exclude change caused by analytic and biologic variations [16]. A correction of NT-pro-BNP values according to age and eGFR may be useful and several studies sought to provide reference values according to the type of HF onset (i.e., acute vs. chronic) and CKD stages, although they are not yet validated in clinical practice [17–19]. The results of these studies are reported in Table 1. Among natriuretic peptides, NT-pro-BNP may be considered the most accurate biomarker, providing further useful prognostic information in patients with HF and CKD, such as cardiovascular events, all-cause death, and quality of life [19].

**Table 1:** Adjusted cut-off values of natriuretic peptides used in HF assessment in patients with CKD

**Biomarker**	**HF type**	**Prognosis**	**Cut-off**	**Cut-off adjusted according to eGFR**
**NT-ProBNP**	AHF/CHF	Short- and long-term cardiovascular events and all-cause death	AHF: 300 pg/mL	*As diagnostic value:*
			CHF: 125 pg/mL	-AHF and CKD stage 3–5: 1200–6000 pg/mL
				*As prognostic value:* Only two studies have tried to give a cut-off:
				-Horii et al. reported 259.7 pg/mL (eGFR > 30 mL/min/1.73 m2) and 5111 pg/mL (eGFR < 30 mL/min/1.73 m2) [17]
				-Masson et al. reported 769 pg/mL (eGFR > 60 mL/min/1.73 m2) and 2023 pg/mL (eGFR < 60 mL/min/1.73 m2) [122].
**BNP**	AHF/CHF	Short- and long-term cardiovascular events, all-cause death, and quality of life.	*Diagnostic threshold:*	*As diagnostic value:*
			AHF > 400 pg/mL	CHF: in only one study was provided a cut-off:
			CHF: > 150 pg/mL	*-*McCullough et al. reported for patient with CKD stages 3–5 (eGFR < 60 mL/min/1.73m2) > 200 pg/mL [123]
				*As prognostic value:*
				CHF: in only one study was provided a cut-off:
				*-*Horii et al. reported 90.8 pg/mL (eGFR > 30 mL/min/1.73 m2) and 157 pg/mL (eGFR < 30 mL/min/1.73 m2) [17]

*AHF* acute heart failure, *CHF* chronic heart failure

The BNP metabolism is mainly non-renal, mediated by the binding to the natriuretic peptide receptor type C (NPR-C) and through proteolysis by neutral endopeptidases; thus, its concentration in CKD patients is less affected by eGFR, without the need of a correction of values in patients with CKD stages 1–2 [20]. In asymptomatic HF patients, the rise of BNP blood levels reflects left ventricular overload, predicting a high risk of symptom development. For patients with CKD and HF, a high BNP strongly predicts cardiovascular events and death from all causes. Moreover, patients with a reduction of BNP after treatment have a better prognosis than those with an increase or stable values of BNP [21].

Given the reduced renal excretion, patients with CKD accumulate toxins, which can cause specific damage to cardiomyocytes. P-cresyl sulfate, a cresol-derived protein-bound toxin, and asymmetric dimethylarginine, a product of protein catabolism in cells, are related to cardiovascular outcome and mortality in recent studies and they may contribute to cardiac hypertrophy and endothelial dysfunction in in vitro experiments [22, 23]. Although promising, the role of these toxins in HF management is not sufficiently specified and validated in clinical practice. These and other toxins could explain elevated biomarkers of myocyte damage as eGFR decreases.

Myocyte damage biomarkers play an important role in the diagnostic and prognostic evaluation of patients with HF and CKD. The increase of high-sensitive troponin T (HsTnT), the most significant biomarker of myocyte injury, is related with the severity of HF and may correlate with poor prognosis in patients hospitalized with HF [21]. In case of CKD and HF, the cut-off value of all-cause death should be adjusted according to eGFR. HsTnT can predict death in CKD patients without cardiac symptoms and may predict the occurrence of HF. As the eGFR decreases, the prediction accuracy of HsTnT slightly decreases, particularly for CKD stage 5 [24] (Table 2).

**Table 2:** Biomarkers of myocardial injury for CKD patients with HF

**Biomarker**	**Prognosis**	**Prediction**	**Cut-off adjusted according to eGFR**
**hsTnT**	Short- and long- term mortality	HF occurrence and death in CKD without cardiac symptoms	As prognostic value: -13 ng/L CKD stage 1 -15 ng/L CKD stage 2 -22 ng/L CKD stage 3 -40 ng/L CKD stages 4–5 As prediction value in patient without HF: <5 ng/L lower risk of HF within 12 years

Renal biomarkers represent additional tools in the diagnostic algorithm. Creatinine and eGFR remain the reference biomarkers for acute and chronic kidney damage. Overt CKD is defined by eGFR < 60 mL/min/1.73 m2 of body surface area, and/or by the presence of albuminuria (high 30–300 mg albumin/1 g of urine creatinine or very high > 300 mg albumin/1 g of urine creatinine) for 3 months or more [25]. Instead, for the diagnosis of acute kidney injury (AKI), there are different criteria, such as KDIGO (Kidney Disease: Improving Global Outcomes) criteria, RIFLE (Risk, Injury, Failure, Loss of kidney function, and End-stage kidney disease), and AKIN (Acute Kidney Injury Network) classifications. According to these, the acute kidney damage is staged based on the severity of the parameters provided. In KIDGO criteria, AKI is classified into 3 stages of severity according to the value of serum creatinine or urinary output. In stage 1 the serum creatinine (SCr) is 1.5–1.9 times the baseline value or the urinary output less than 0.5 mL/kg/h for 6–12 h. The stage 2 is characterized by a SCr between 2.0 and 2.9 times the baseline value or a urinary output less than 0.5 mL/kg/h for more than 12 h. In presence of a more severe AKI (stage 3) the SCr is more than 3.0 times the baseline value, or an absolute value over 4.0 mg/dL, or a renal replacement therapy was initiated or in case of a more severe decrease of urinary output (less than 0.3 mL/kg/h for 24 h or anuria for more than 12 h) [26].

Novel clinical biomarkers reflecting glomerular and tubular injury are currently available and they are listed in Table 3. In a subset of patients with HF, serum cystatin C (CysC), which is a marker of glomerular filtration rate, together with albuminuria, a marker for CKD severity, could be a strong predictor of rehospitalization and short- and long-term mortality. Plasma CysC can be used to estimate eGFR, which has been shown to be in good agreement with eGFR calculated from inulin clearance [27]. In patients with CKD and HF, several studies report that CysC may provide more prognostic information than creatinine and it can detect more correctly the risk groups of all causes of death and recurrent HF, although a clear cut-off value has not been yet proposed [28, 29]. Its plasma concentrations are influenced by smoking habit, cancer, thyroid diseases, obesity, and it is not routinely used in clinical practice. Recent studies highlight that excessive increase in plasma CysC can promote myocardial fibrosis through the accumulation of osteopontin and TIMP-1, and it can promote atrial dilatation and ventricular hypertrophy, resulting in diastolic dysfunction [30]. Neutrophil gelatinase-associated lipocalin (NGAL), in patient with CKD and acute heart failure (AHF), has a diagnostic and prognostic value [31]; the combination of tissue inhibitor of metalloproteinases-2 (TIMP-2) and insulin-like growth factor binding protein 7 (IFGBP7) is a useful diagnostic and prognostic biomarker in AKI [32].

**Table 3:** Novel promising clinical biomarkers reflecting glomerular and tubular injury

**Biomarker**	**Prediction**	**Characteristics**
**Serum cystatin C (CysC)**	Strong independent predictor of rehospitalization and mortality in CRS	Markers of glomerular filtration and integrity also promote myocardial fibrosis and promote atrial dilatation and ventricular hypertrophy, resulting in diastolic dysfunction [26–28].
**Neutrophil gelatinase-associated lipocalin (NGAL)**	Serial measurements increase its predictive value for AKI	It is a protein found in neutrophil granules that is secreted by renal tubular epithelium and represent a marker of renal tubular injury.
**The combination of inhibitor of metalloproteinases-2 (TIMP-2) and insulin-like growth factor binding protein 7 (IFGBP7)**	Useful diagnostic tool in AKI setting	Both tubular biomarkers involved in G1 cell cycle arrest during the early phase of cell injury. They correlated with congestion and may play a role in AHF and guide decongestive therapies [124].

*AKI* acute kidney injurym *CRS* cardio-renal syndrome, *AHF* acute heart failure

Clinical data in CKD patients identified circulating fibroblast growth factor 23 (FGF23) as a marker for the diagnosis and prognosis of HF in CKD. FGF23 is a hormone produced by osteocytes that inhibits phosphate reabsorption and 1,25(OH)2D synthesis in the kidney. Multiple studies show that FGF23 production progressively increases during CKD and its blood concentrations are associated with incident HF and cardiovascular events in general population and CKD patients [33–35]. Mechanisms supporting these clinical observations are explained by findings in in vitro experiments showing that FGF23 may stimulate myocardial hypertrophy through a direct effect on cardiomyocytes [36, 37] and fibroblasts leading to cardiac remodeling and fibrosis [38, 39]. In addition, the activation of RAAS and the administration of angiotensin II are able to increase FGF23 secretion by osteocytes in mice [38]; accordingly, patients with normal kidney function show an association of serum FGF23 with serum levels of BNP and cardiovascular adverse events that decreased in patients taking ACEIs [40]. Thus, FGF23 may be an independent predictor of cardiovascular risk in CKD patients [41] and the use of serum FGF23 determination in the clinical practice has been suggested, but not currently established, as its diagnostic and clinical value yet needs to be better detailed in prospective studies.

Canonical effects of FGF23 in the kidney may be modulated by Klotho, a membrane protein working as a cofactor of FGF23 receptor. The extracellular domain of Klotho may be cleaved and released in blood as soluble Klotho that may have protective effects on heart and arteries [42, 43]. Serum levels of soluble Klotho are associated with a lower risk of HF in a cross-sectional analysis of National Health and Nutrition Examination Survey (NHANES) population [44]. Beneficial effects of Klotho are lost in CKD as its serum levels decrease with the decline of GFR. Therefore, soluble Klotho could be considered to define the weight of serum FGF23 for the diagnosis of HF in CKD.

As mentioned above, clinical standards for the prognosis of HF depend on protein-based biomarkers. Recently, new opportunities for genetic analysis are emerging as a new approach to understand the pathophysiology of HF and cardiovascular disease, paving the way for the development of gene-based biomarkers. “Omics” technology, which identifies gene variations at the genome and transcriptome levels, is a novel approach to identify DNA/RNA-based biomarkers [45, 46]. In addition, omics analysis not only enables the identification of genetic variations that may contribute to the identification of HF risk but also provides insight into the molecular mechanisms of the underlying disease. Although the application of new genetic biomarkers, such as genetic risk scores in disease prognosis, promises to improve disease risk estimation, unfortunately, only limited studies are available, and the efficacy of most new biomarker candidates has yet to be demonstrated [47]. Although fascinating, the true value of these new potential biomarkers in the prognostic assessment of HF remains unclear.

## Imaging modalities

Echocardiography is an imaging modality which play a key role in the diagnostic algorithm of suspected HF. It enables the identification of underlying anatomical and functional abnormalities and it is useful to define the etiology of HF, through the evaluation of cardiac chamber size, wall thickness, and the diastolic-systolic function (global and regional qualitative assessment, quantitative volume estimation,), the right chamber assessment, valve function, and pericardial and endocardial integrity [48–51]. Moreover, the assessment of the left ventricular ejection fraction (LVEF) is necessary for the classification of HF into three phenotypes: HFpEF with a preserved systolic function (i.e. LVEF > 50%), HFmrEF with a LVEF between 41 and 49%, and HFrEF with a LVEF < 40%. This classification is of utmost importance in order to guide the therapeutic approach. LVEF is also fundamental for the decision-making regarding devices therapy such as implantable cardioverter–defibrillator (ICD) or cardiac resynchronization therapy–defibrillator (CRT-D) [52].

Furthermore, an echocardiographic re-evaluation during the follow-up is required in case of a change in the clinical picture. Additional techniques such as tissue Doppler imaging (TDI) and strain rate (SR) should be considered to identify patients who are at risk of HF or to identify early worsening of HF [53, 54]. Hassanin et al. have shown that in patients with CKD, left ventricle (LV) longitudinal systolic strain and early and late diastolic strain rates are significantly reduced despite a preserved LVEF, with an early identification of patients at high risk of developing HF [55]. Krishnasamy et al. have demonstrated that global longitudinal strain predicts mortality for all cause in the presence of CKD [56].

**Fig. 1:**
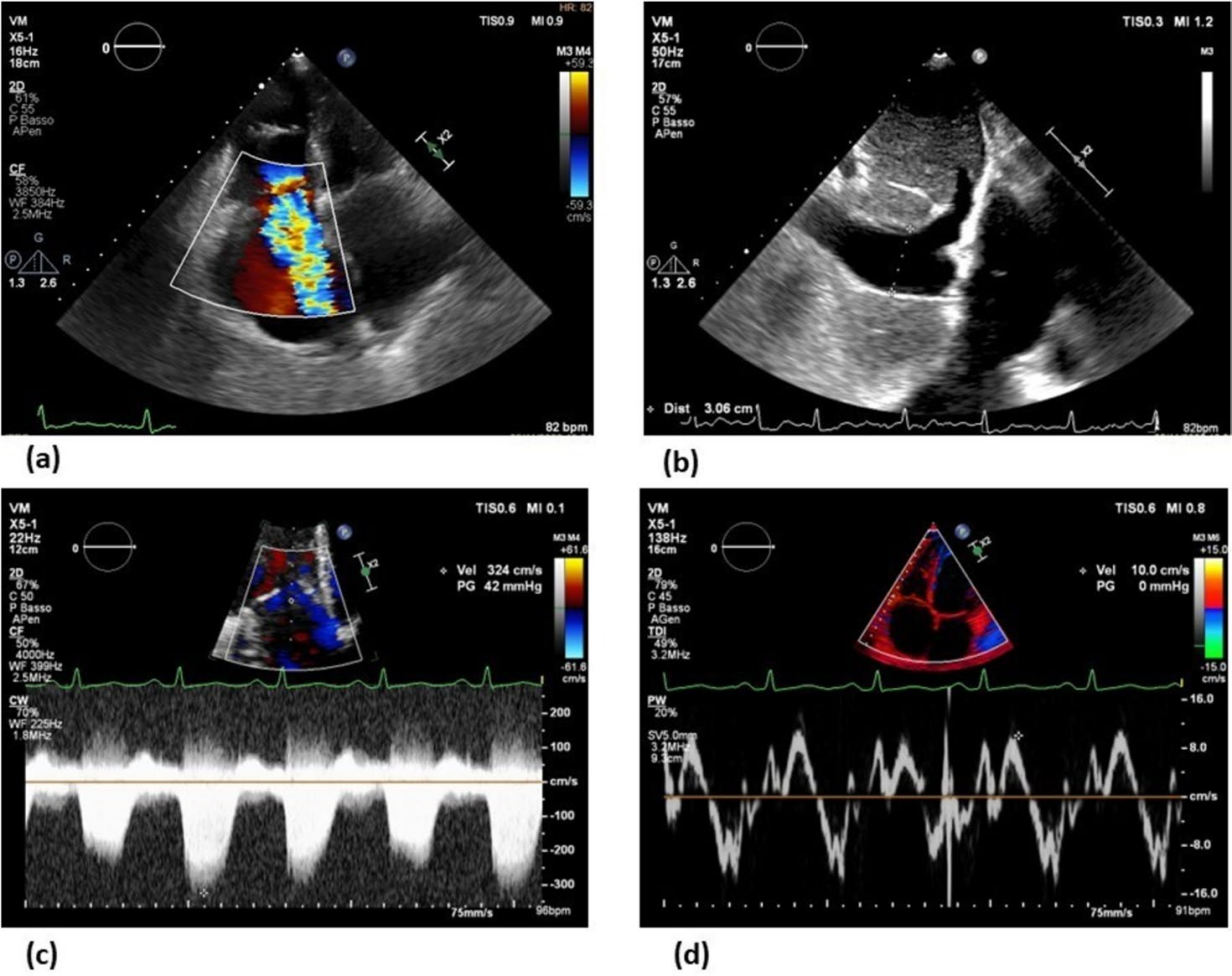
Echocardiographic evaluation of the volume status in heart failure (HF). A case of a patient with HF and fluid overload at the echocardiographic examination, requiring a prompt diuretic treatment. **a** The bidimensional assessment in the four-chamber view shows a dilated right ventricle, with a severe tricuspid regurgitation and an enlarged right atrium. **b** The subcostal view displays a marked dilation of the inferior vena cava with a reduced inspiratory collapse. **c** The continuous wave Doppler on the tricuspid regurgitations may allow an estimation of pressures in the right heart chambers. **d** Peak systolic velocity on tricuspid annulus by tissue Doppler imaging (S′ wave) represents a useful tool to assess the systolic function of the right ventricle

Echocardiography is also required to estimate the volume status, with a prompt detection of patients with venous congestion and fluid overload, guiding the diuretic therapy. In this contest, several echocardiographic parameters should be considered: the right ventricular systolic function using the fractional area change (FAC), the tricuspid annular plane systolic excursion (TAPSE) or the peak systolic velocity on TDI (S′ wave) [57], the maximum tricuspid valve regurgitation jet velocity [58, 59], and the diameter and decreased inspiratory collapse of inferior vena cava, which together are helpful for the estimation of the right chamber pressures. These parameters are shown in Fig. 1. A reduced pulmonary valve acceleration time, a dilated pulmonary artery, and an enlarged right atrium are parameters related to pulmonary capillary wedge pressure that can be used to assess the hemodynamic status [60, 61].

In patients with HF, the dilation of the renal veins with a slow and continuous flow can be observed. Renal ultrasound, furthermore, gives information on the chronicity of the disease by the kidney size, cortical echogenicity, and abnormal cortical-medullary ratio, so it is useful in identifying the progression of CKD in HF patients [62]. In addition, Doppler measurement of intrarenal venous flow is useful for the estimation of right atrial pressure [63].

In CKD an echocardiographic evaluation is required in the pretransplantation work up for the screening of cardiovascular diseases in high-risk patients before kidney transplantation [64]. KDIGO Clinical Practice Guidelines on the Evaluation and Management of Candidates for Kidney Transplantation suggest an echocardiographic assessment in asymptomatic candidates on dialysis for at least 2 years or with risk factors for pulmonary hypertension (e.g., portal hypertension, connective tissue disease, congenital heart disease, chronic obstructive pulmonary disease) [65]. Moreover, echocardiography is recommended by K/DOQI clinical practice guidelines for cardiovascular disease in dialysis patients in case of end-stage kidney disease at the initiation of hemodialysis, once patients have achieved dry weight (ideally within 1–3 months of dialysis initiation), and every 3 years thereafter [66, 67].

Cardiac magnetic resonance (CMR) represents the “gold standard” imaging technique and it is a valuable resource for the morpho-functional study in patients with poor acoustic window. Gadolinium-based contrast agents (GBCA) may be required for the identification of cardiac fibrosis, although there are concerns regarding their use in case of kidney impairment, due to the higher risk of adverse side effects such as nephrogenic systemic fibrosis. In patients with mild-to-moderate CKD, the administration of standard doses of GBCA is safe without the need of further precautions. In the presence of AKI or a severe kidney impairment (i.e., eGFR < 30 mL/min/1.73 m2) or dialysis, there are no strict contraindication to their use, although the decision should be made in a case-by-case manner according to the risk/benefits ratio and alternative imaging modalities should be preferred, whenever possible [68].

## Toward personalized treatment

The therapeutic management of patients with HF and CKD remains difficult due to several reasons. Indeed, kidney dysfunction is a typical exclusion criterion in randomized clinical trials (RCT), leading to a lack of adequate evidence on the use of guideline-directed medical therapy (GDMT) in HF patients with a concomitant CKD. Moreover, altered drug pharmacokinetics and plasma electrolyte abnormalities may hamper their efficacy, increasing the risk of adverse side effects. Hence, the treatment strategy should be planned considering the phenotype of HF and the degree of CKD, with a multidisciplinary approach, and providing a close clinical and laboratory monitoring of the patient.

During the last years, several large RCT have revolutionized the therapeutic scenario of HFrEF, while data for HFmrEF and HFpEF phenotypes are less consistent.

ACEIs, angiotensin receptor blockers (ARBs) and mineralocorticoid receptor antagonists (MRAs) are recommended to reduce cardiovascular and all-cause mortality in patients with HFrEF [69]. In case of CKD stages 1–3, they should be used providing a close monitoring of creatinine and potassium. In patients with HFrEF and CKD stages 4 and 5, they may be used with caution and adjusting the dose if necessary. There is limited evidence of their clinical efficacy in patients on dialysis. Recent data from the STOP ACEi trial, a multicenter, randomized, open-label trial in 411 patients with advanced chronic kidney disease suggest that the discontinuation of RAS inhibitors was not associated with a significant changes in the eGFR, rate of serious adverse cardiovascular, vascular, and heart-failure events in the long term follow up (3 years), as compared to patients continuing RAS inhibitors treatment [70]. A post hoc analysis of the Study of Left Ventricular Dysfunction (SOLVD) demonstrated a reduction of mortality even in the presence of higher degrees of CKD in HF patients treated with ACEIs [71]. Although an impairment of kidney function can occur after starting ACEIs, there are long-term advantages. In fact, the reduced hospitalization rate and benefit on survival justify the continuation of therapy to an increase up to 35% of creatinine [72]. Hyperkalemia is not rare as a side effect, requiring their interruption or a dose reduction. In addition, in patients with HF and CKD, serum potassium levels may fluctuate as a result of dietary changes and an inappropriate use of diuretics. Oral potassium binders, such as patiromer or sodium zirconium cyclosilicate, may be a valid option to avoid the withdrawal or dose reduction of disease modifying drugs with an influence on serum potassium levels [73]. Patiromer may prevent hyperkalemia in normokalemic HF patients with or without CKD treated with MRAs as demonstrated in a randomized, double‐blind, placebo‐controlled study [74].

Moreover, MRA molecules with less affinity to other steroid receptors, such as finerenone and eplerenone, should be preferred, due to less influence on potassium levels and reduced systemic adverse effects like gynecomastia, low libido, and impotence, often leading to the discontinuation of MRA therapy [75, 76].

MRAs when added to an ACEI/ARB can provide additional suppression of RAAS with potential long-term cardiorenal benefits. The reduction of mortality, hospitalization, and cardiovascular events in HFrEF has been pointed out in several studies such as Randomized Aldactone Evaluation Study (RALES) [77] and Eplerenone in Mild Patients Hospitalization and Survival Study in Heart Failure (EPHASIS-HF) [78]. These studies include patients with CKD (GFR 30–60 ml/min per 1.73 m^2^) with safe and effective outcomes. Data on the safety and efficacy of MRAs in HFrEF with advanced CKD (stage 4 and 5) are limited [79].

Several RCT have reported a reduction of cardiovascular and all-cause mortality in patients with HFrEF treated with angiotensin receptors-neprilysin inhibitors (ARNI) as compared to ACEIs/ARBs [80]. Accordingly, the American College of Cardiology (ACC)/American Heart Association (AHA) and the European Society of Cardiology (ESC) recommend ARNI as first-line treatment of HFrEF when patients remain symptomatic despite optimal treatment with ACEIs or ARBs. Potential benefits on kidney function are observed even in moderate CKD (i.e., eGFR between 30 and 60 mL/min/1.73 m^2^), with a reduced decline of eGFR, with a low rate of hyperkalemia [81]. Although, most of large RCT excluded patients with a severe reduction of kidney function, Haynes et al. have demonstrated safety and efficacy of ARNI in patients with an eGFR lower than 20 mL/min/1.73 m2 [82]. In case of an eGFR lower than 60 mL/min/1.73 m^2^, the starting dose should be 24/26 mg bis in die, with a cautious up-titration under close monitoring of blood creatinine, potassium levels, and arterial blood pressure.

β-blockers are another cornerstone of GDMT, due to the demonstrated reduction of mortality and morbidity in HFrEF patients. They are strongly recommended for HFrEF by guidelines [52]. Although the lack of studies on large populations, they seem to be safe and effective also in CKD patients [83, 84]. Carvedilol has been shown to be beneficial in patients with HF and CKD stage 5 and improves mortality in HFrEF patient on hemodialysis [85]. Thus, it should be preferred due to the absence of dialytic clearance and the reduced risk of hyperkalemia as compared to other beta blockers [86].

Studies on ivabradine, approved for HFrEF as second-line therapy, include patients with eGFR < 60 mL/min/1.73 m2 and the benefits are the same as compared to patients without kidney dysfunction [87].

Sodium-glucose cotransporter inhibitors 2 (SGLT2Is) represents the latest drug approved as a first-line treatment for HFrEF and recommended by the guidelines irrespective of diabetic status [69]. SGLT2Is reduce cardiovascular mortality and hospitalization [i.e., dapagliflozin: HR 0,74 (95% Cl 0,65; 0,85), *p* < 0.0001]. The Dapagliflozin in Patient with Chronic Kidney Disease (DAPA-CKD) trial is an international, multicenter, randomized, double-blind, placebo-controlled study in patients with CKD (eGFR > 25 mL/min per 1.73 m^2^ but < 75 mL/min per 1.73 m^2^ and proteinuria ≥ 200 and ≤ 5000 mg/g). Dapagliflozin is superior in preventing a sustained ≥ 50% drop in eGFR, development of end-stage renal disease, and cardiovascular or renal death. In patients with CKD, treatment with dapagliflozin improves overall survival, with a significant reduction in all-cause mortality [88]. In the recent trial EMPEROR-reduced, a significant reduction (25%) of cardiovascular death and HF hospitalizations has been observed in patient treated with empagliflozin including those with eGFR as low as 20 mL/min per 1.73 m^2^. The eGFR decline is slower with empagliflozin as compared with placebo [89]. Monitoring of kidney function using eGFR and blood creatinine levels is not standardized. Discontinuation or dose reduction is not currently suggested in case of a mild decrease of eGFR (3–4 mL/min/1.73 m^2^), encountered very frequently in the first 2–3 weeks after the initiation of SGLT2Is therapy. The aforementioned drugs, including SGLT2Is, may often cause a mild initial but transient decrease of eGFR; thus, their withdrawal is not usually required. An increase in serum creatinine of lower than 50% above baseline provided it is < 266 μmol/L (3 mg/dL) or a decrease in eGFR lower than 10% of baseline provided eGFR is > 25 mL/min/1.73 m^2^ may be acceptable, because, in the long-term follow up, SGLT2Is and other drugs are capable to slow the decline of eGFR, reduce proteinuria, and preserve kidney function compared to placebo. For these reasons, these drugs should not be discontinued without a clear contraindication [90] (Table 4).

**Table 4:** Disease-modifier medical therapy of HF available adjusted for renal function

**Drug available**	**Recommended CREATININE (mg/dL) or eGFR (mL/min/1.73 m**^**2**^**)**
Angiotensin-converting enzyme inhibitors (ACEIs)	< 3.4 mg/dL (enalapril) [125]
Angiotensin receptor blockers (ARBs)	< 3.4 mg/dL (valsartan) [126]
Mineralocorticoid receptor antagonists (MRAs)	< 2.5 mg/dL (spironolactone) [127] > 30 mL/min/1.73 m^2^ (eplerenone) [79]
β blockers	< 3.4 mg/dL (bisoprolol) [128]
Vericiguat	≥ 15 mL/min/1.73 m^2^ [129]
Hydralazine/isosorbide dinitrate	No restrictions [130]
Angiotensin receptor neprilysin inhibitor (ARNI)	> 30 mL/min/1.73 m^2^ [131]
Ivabradine	< 2.5 mg/dL [87]
SGLT2Is	
Dapagliflozin	> 30 mL/min/1.73 m^2^ [132]
Empagliflozin	> 20 mL/min/1.73 m^2^ [89]

A recent study also has revealed that in a population of patients with different causes of CKD, with different levels of eGFR (even below 30 mL/min/1.73 m2), empagliflozin safely reduces the progression of renal disease and death from cardiovascular causes of about 28%. The risk of hospitalizations for any cause is reduced by 14%. These results suggest that SGLT2Is may be a prognostically relevant drugs also in patients with advanced renal disease [91].

Diuretic therapy is a mainstay for cardiac congestion. However, the efficacy decreases as the kidney function declines; thus, it is difficult to optimize the treatment in these patients. High doses of intravenous loop diuretic, oral metolazone, oral loop diuretic, and oral thiazide diuretic (though often ineffective in stages 4 and 5 of CKD) can be used [92]. A recent multicenter, double-blind, randomized, placebo-controlled trial, in 519 patients with acute decompensated heart failure and clinical signs of volume overload with elevated levels of N-terminal pro-B-type natriuretic peptide, points out that the addition of intravenous acetazolamide to standardized intravenous loop diuretic therapy was associated with successful decongestion as compared to placebo (risk ratio, 1.46; 95% confidence interval [CI], 1.17 to 1.82; *p* < 0.001) [93]. Thiazide diuretics influence the reabsorption of the electrolyte in the distal renal tubule, inhibiting the Na/Cl+ transporter, promoting also the reabsorption of ions Ca^++^, increasing loss of urinary bicarbonate and serum potassium levels. Instead loop diuretics act on the Na–K–Cl cotrasporter located in the luminal side of tubular cells in the ascending limb of the loop of Henle. In case of diuretic resistance, the use of different types of diuretics, acting in several segments of the nephron, may be a useful strategy to control symptoms and improve survival. In selected cases, despite the increase in creatinine, aggressive diuresis can be helpful in terms of clinical benefit [94]. Several studies demonstrate that a reduced diuretic response (DR) is associated with an increased risk of death [95]. Other studies have tried to calculate the DR as the ratio of the change in body weight and the amount of furosemide administered, proving the association with patient severity and outcomes [96].

The benefits of cardiac resynchronization therapy in patients with eGFR < 60 mL/min per 1.73 m^2^ are comparable to individuals with normal kidney function [97]. Furthermore, sudden cardiac death rates are higher in patients with advanced CKD and HF [98]. However, in patient with end-stage kidney disease on hemodialysis, the implantation of transvenous devices may be problematic, due to several issues related to the venous limited capital, the significant implant bleeding risk, and the higher risk of device infection [99, 100]. In particular, the transvenous lead may provoke central vein stenosis causing a failure of the arteriovenous (AV) fistula, especially when placed ipsilateral [101]. These concerns may be overcome with the implantation of leadless devices such as subcutaneous ICD or Micra pacemakers, and published studies demonstrate their safety and efficacy in hemodialysis patients [102, 103]. As regard patients with HFpEF, to date, there are no specific treatment options demonstrating a reduction of mortality and morbidity in large populations. Only diuretics are strongly recommended by guidelines to reduce symptoms of congestion [52]. Moreover, great importance is given to the treatment of cardiovascular risk factors and coexisting comorbidities (e.g., hypertension, diabetes mellitus, coronary artery disease, valvular heart disease, iron insufficiency, and anemia). Accordingly, also in the phenotype HFmrEF there are no specific trials exploring the prognostic role of disease-modifying drugs. Treatment with ACEI/ARB/MRA/β-blockers may be considered although diuretic therapy is the only with a strong recommendation to control symptoms [52]. In Table 5 are summarized the HF medications recommended by the latest European guidelines [52] according to the phenotype of HF and the CKD stages. The classical stepwise therapeutic approach of the previous guidelines was abandoned in favor of a timely use of GDMT, with a faster initiation and up-titration of disease-modifier drugs simultaneously, in order to provide their benefits as soon as possible [52]. However, in CKD patients it should be advisable a more cautious approach to prevent side effects and increase the tolerability of these drugs. Based on the result of the aforementioned studies, in HF patients with moderate kidney impairment, the initiation with nephroprotective agents such as SGLT2Is and ARNI should be encouraged. In patients with advanced CKD or dialysis, the therapeutic options are scarce and beta blockers should be considered first option, unless contraindicated.

**Table 5:** Medical therapy recommended by the latest European guidelines [52] in HF according to the phenotype and the CKD stage

**Stages of chronic kidney disease (KDOQI)**					
**HF phenotype**	**Stage 1** **Normal** **GFR ≥ 90 mL/min/1.73 m2)**	**Stage 2** **Mild** **GFR 60–89 mL/min/1.73 m2)**	**Stage 3** **Moderate** **GFR 30–59 mL/min/1.73 m2)**	**Stage 4** **Severe** **GFR 15–29 mL/min/1.73 m2)**	**End-stage renal disease** **GFR < 15 mL/min/1.73 m2) or dialysis**
**HFrEF**	*BB*	*BB*	*BB*	*BB*	
	*ACE-I/ARB -ARNI*	*ACE-I/ARB -ARNI*	*ACE-I/ARB -ARNI*	*ACE-I/ARB*	
	*MRA*	*MRA*	*MRA*		
	*SGLT2i*	*SGLT2i*	*SGLT2i*	*SGLT2i*^a^	
	*Diuretics*	*Diuretics*	*Diuretics*	*Diuretics*^b^	
	**Ivabradine**	**Ivabradine**	**Ivabradine**	**Ivabradine**	
	Hydralazine/isosorbide dinitrate	Hydralazine/isosorbide dinitrate	Hydralazine/isosorbide dinitrate	Hydralazine/isosorbide dinitrate	Hydralazine/isosorbide dinitrate
	Vericiguat	Vericiguat	Vericiguat	Vericiguat	
	Digoxin	Digoxin	Digoxin	Digoxin	
**HFmrEF**	*Diuretics*	*Diuretics*	*Diuretics*	*Diuretics*^b^	
	ACE-I/ARB – ARNI	ACE-I/ARB – ARNI	ACE-I/ARB – ARNI	ACE-I/ARB	
	MRA	MRA	MRA		
	BB	BB	BB	BB	
**HFpEF**	*Diuretics*	*Diuretics*	*Diuretics*	*Diuretics*^b^	

Italic: the drug is recommended (class I of recommendation), Bold: the drug should be considered (class IIa), Underline: the drug may be considered (class IIb)

*HFrEF* heart failure with reduced ejection fraction, *HFmrEF* heart failure with mildly reduced ejection fraction, *HFpEF* heart failure with preserved ejection fraction, *GFR* glomerular filtration rate, *BB* β blockers, *ACE-I* angiotensin converting enzymes inhibitor, *ARB* angiotensin-receptor blocker, *ARNI* angiotensin receptor-neprilysine inhibitor, *MRA* mineralcorticoid receptor antagonist, *SGLT2i* sodium glucose cotrasporter-2 inhibitor, *KDOQI* kidney disease outcomes quality initiative

^a^Only empagliflozin in case of GFR between 30 and 20 mL/min/1.73 m2

^b^Loop diuretics are preferred to thiazide diuretics

## Peritoneal dialysis in refractory congestive heart failure

Peritoneal dialysis (PD) may be considered a treatment option in patients with refractory volume overload unresponsive to diuretic treatment [69].

Recent studies including patients with refractory HF suggest a positive effect of PD on the functional status, hospitalization rate, and quality of life while, at the moment, no advantage has been shown on survival with a significant pooled mortality rate of 37% [104]. An improvement of functional New York Heart Association (NYHA) classification for patients treated with peritoneal ultrafiltration (PUF) was observed for patients surviving the first 6 months, with an improvement of functional status from NYHA IV to NYHA II in about one half of cases, although a higher mortality rate was noted for patients starting with a NYHA class IV [105]. Several studies evaluating the clinical effects of PD in patients with diuretic resistant HF showed a significant reduction in hospitalization rates for both the number of admission and days spent in hospital [106]. A recent meta-analysis of Timoteo et al. reports a significant decline in hospitalization days by almost 35 days/patient/year [104]. An improved quality of life according to Minnesota Living with Heart Failure Questionnaire is found when compared with quality of life measured before starting of dialysis [107].

PUF allows a restoration of sensitivity to diuretics and a rise of urine output connected to an improvement in cardiac performance [108]. A slight but statistically significant (*p* < 0.02) improvement of about 5% from the baseline of the LVEF is observed [104]. This favorable effect may be related to fluid extraction, which lead to the movement of the Frank-Starling curve to left [109] or the removal of myocardial depressant toxins [110]. Moreover, the drainage of ascites might decrease intraperitoneal pressure which has been demonstrated to improve kidney function in HF [111].

There are insufficient data regarding in which patient PUF may be beneficial. Patients eligible for PUF must have impaired kidney function (eGFR < 50 mL/min/1.73 m^2^: stage 3 CKD of the KDOQI classification) [112]. Potential candidates for PUF are those unresponsive to diuretic treatment, with frequent hospital admission for decompensated HF and not eligible for VAD/transplantation [69].

Severe complications are rare and the most common is peritonitis with a rate of 0.26–0.37 episodes/patient/year, mainly when PD is used in the ambulatory setting [113–115]. The main clinical contraindications for PUF include abdominal inflammatory process as ulcerative colitis and Crohn’s disease, end-stage liver diseases, the presence of ostomies, and unrepaired hernias [116].

Residual kidney function influences the peritoneal dialysis prescription. In case of adequate residual kidney function but need for fluid removal, all low dose PD regimens (incremental PD) can be used to obtain adequate PUF. Instead, patients with inadequate residual kidney function and need for solute and fluid removal require full dose PD. Incremental PD regimens consist in a reduced number of dialysate exchanges and can be prescribed in manual continuous ambulatory peritoneal dialysis (CAPD) with 1 or 2 dwell periods/day or in automated peritoneal dialysis (APD) with 3–4 session/week [117].

In PD water and solute are removed over the peritoneal membrane by dwelling dialysate solution in the peritoneal cavity. Dialysate solution by an osmotic gradient drive peritoneal UF while convective and diffusive forces induce solute removal, including the removal of sodium and potassium [118]. Crystalloid and colloid dialysate solutions are currently available. Crystalloid solutions are dextrose or amino acid based and they induce solute-free water transport across the water channels of peritoneal membrane. Colloid osmosis, induced by a mixture of glucose polymers (maltodextrins) called icodextrin, does not induce free water transport but UF with solutes. Icodextrin allows for more sodium removal compared to an equal UF volume induced with a dextrose-based solution. Moreover, the UF volume is higher than conventional dextrose solutions, although with a lower rate of ultrafiltration at the onset of dialysis but sustained over a longer period (8–12 h) [119, 120].

Both CAPD and APD regimens have been proposed to treat fluid overload in HF. In patient with significant residual kidney function, as first regimen a single manual night-time icodextrin exchange, which is able to maintain slow and constant UF during long dwells, can be used. If clinically required, PUF can be increased with two exchanges/day using glucose, at concentrations that vary according to the UF obtained, plus a night-time exchange with icodextrin. Alternatively, APD, that uses a machine for exchanges (cycler), can be used up to 3–4 sessions/week using different concentrations of glucose in night-time and icodextrin in daytime. Patients with HF and advanced stage 5 CKD require full dose PD regimens that include CAPD with 3–5 dwell periods/day or daily APD [121].

## Conclusions

Many clinical studies have shown that most of drugs indicated as first line treatment by guidelines for HF are effective in improving patient prognosis and they can also be used in patients with mild to moderately impaired kidney function. Many of these studies have mostly excluded patients with advanced chronic kidney failure (eGFR < 20–30 mL/min/1.73 m^2^) with less safety per treatment in this group. Therapy for HF in patients with CKD remains difficult, poorly demonstrated, poorly established and standardized, but nevertheless undergoing research. Future studies should address patients with advanced chronic kidney failure and kidney replacement therapy to facilitate their management and support the clinician in therapeutic choices.

## Data Availability

No new data were created for this review.

## Abbreviations

HF: Heart failure
CKD: Chronic kidney disease
eGFR: Estimated glomerular filtration rate
HR: Hazard ratio
CI: Confidence interval
CRS: Cardio-renal syndrome
HFpEF: Heart failure with preserved ejection fraction
HFrEF: Heart failure with reduced ejection fraction
HFmrEF: Heart failure with mildly reduced ejection fraction
RAAS: Renin-angiotensin aldosterone system
BNP: B type natriuretic peptide (BNP)
NT-pro-BNP: N-terminal pro-B type natriuretic peptide
HsTnT: High-sensitive troponin T
AKI: Acute kidney injury
CysC: Cystatin C
NGAL: Neutrophil gelatinase-associated lipocalin
AHF: Acute heart failure
TIMP-2: Tissue inhibitor of metalloproteinases-2
IFGBP7: Insulin-like growth factor binding protein 7
FGF23: Fibroblast growth factor 23
ACEIs: Angiotensin-converting enzyme inhibitors
LVEF: Left ventricular ejection fraction
ICD: Implantable cardioverter defibrillator
CRT-D: Cardiac resynchronization therapy defibrillator
TDI: Tissue Doppler imaging
SR: Strain rate
LV: Left ventricle
FAC: Fractional area change
TAPSE: Tricuspid annular plane systolic excursion
GBCA: Gadolinium-based contrast agents
RCT: Randomized clinical trials
GDMT: Guidelines-directed medical therapy
ARBs: Angiotensin receptor blockers
MRAs: Mineralcorticoid receptor antagonists
ARNI: Angiotensin receptors-neprilysin inhibitors
SGLT2Is: Sodium-glucose cotransporter inhibitors 2
DR: Diuretic response
AV: Arteriovenous
PD: Peritoneal dialysis
NYHA: New York Heart Association
PUF: Peritoneal ultrafiltration
CAPD: Continuous ambulatory peritoneal dialysis
APD: Automated peritoneal dialysis
